# First person – Nienke Zwaferink

**DOI:** 10.1242/bio.062471

**Published:** 2026-01-29

## Abstract

First Person is a series of interviews with the first authors of a selection of papers published in Biology Open, helping researchers promote themselves alongside their papers. Nienke Zwaferink is first author on ‘ Thermal window of exercise performance of the ecosystem engineer *Lanice conchilega*’, published in BiO. Nienke conducted the research described in this article while a Master's student in Professor David Thieldges's lab at the Royal Netherlands Institute for Sea Research, The Netherlands, and the Groningen Institute for Evolutionary Life Sciences, The Netherlands. She is now a recently graduated Master's student interested in the mechanisms by which marine benthic species act as habitat-formers and influence the structure and functioning of coastal ecosystems.


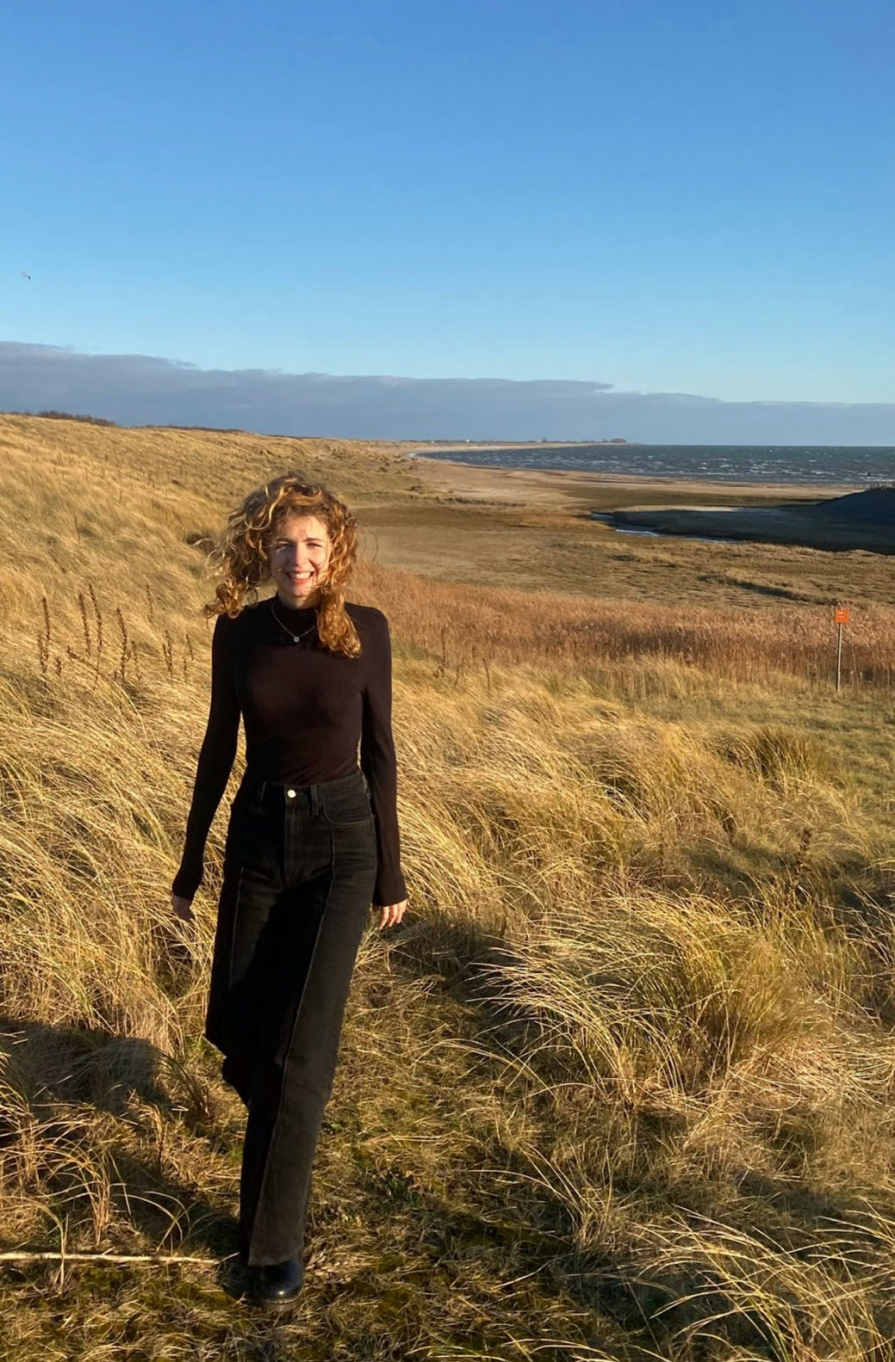


**Nienke Zwaferink**

**Describe your scientific journey and your current research focus**

Just three months ago, I completed my Master's degree in marine biology. The publication in BiO is based on the findings from my first Master's research project at the Royal Netherlands Institute for Sea Research. Between completing this project and preparing it for publication, I travelled to Australia for my second and final research project, studying benthic invertebrates and the interactions between mangroves and oysters. Since graduation, I have been working at my university as a research and teaching assistant. At the same time, I am already looking ahead for opportunities to continue my academic career.

**Who or what inspired you to become a scientist?**

I was inspired by the female scientists I met during my studies. One example is Professor Laura Govers, whose efforts contributed to the successful restoration of intertidal seagrass meadows in the Wadden Sea. Beyond her scientific achievements, she emphasises the social aspects of research by engaging with stakeholders, communicating findings to the public, and highlighting the societal relevance of ecological work. She is just one of many female scientists who have inspired me. These women's passion for research was contagious and redefined my perception of ‘ivory tower’ scientists, motivating me to pursue a scientific career of my own.

**How would you explain the main finding of your paper?**

In this Research Article, we studied the tube-building worm *Lanice conchilega*, a species that lives in sandy coastal areas. By building upright tubes in the seabed, it protects itself and creates habitat for other organisms. In high densities, the tubes form reef-like structures that modify the environment, making it more suitable for a wide range of species to feed or live in. Despite its importance, little is known about how much warming or cooling this worm can tolerate. Here, we present a novel experimental approach to measure its temperature sensitivity by tracking its ability to build new tube segments. This behaviour is essential for both survival and habitat formation. Understanding how this tolerance changes with the seasons is important because extreme winters or summers could weaken this species and affect the many animals that depend on it.

**What are the potential implications of this finding for your field of research?**

Our study provides a first reference for understanding how temperature influences tube-building in *L. conchilega*. On a larger scale, the formation of tubes enables *L. conchilega* to shape its environment by creating complex habitats that support many other species in the intertidal Wadden Sea. For representing one of the main hotspots of biodiversity in the world, the Wadden Sea has received a UNESCO World Heritage Status. Climate change makes the Wadden Sea environment harsher for many of these species, but *L. conchilega* may represent a silver lining. In a previous study by my supervisors, they found that increases in winter temperature promote the abundance of *L. conchilega*, with cascading positive effects on species richness due to its role as a habitat-forming species (de la Barra et al., 2025). Future studies could apply the method we describe to determine whether *L. conchilega* is more limited by cold winter conditions or by extreme summer heat. Given its key role in structuring intertidal habitats and promoting biodiversity, such experiments could provide valuable insights into how temperature-driven changes in the abundance or distribution of *L. conchilega* may affect the Wadden Sea intertidal community under current and future climate scenarios.

**Figure:**
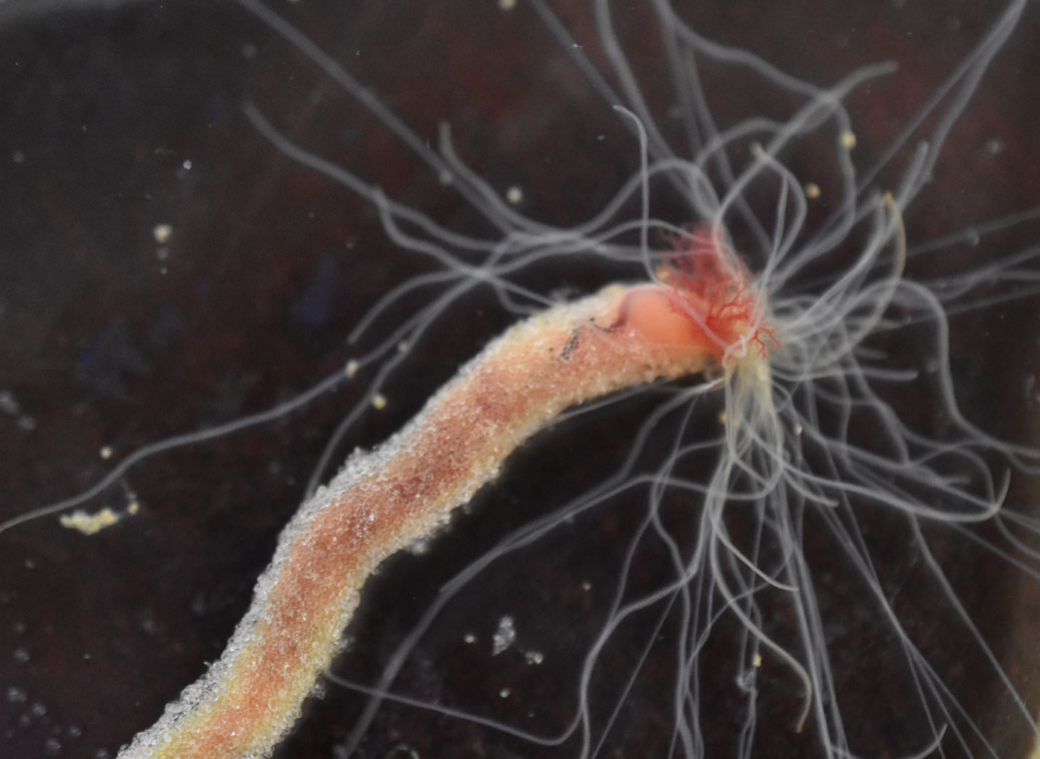
Close-up picture of a *Lanice conchilega* worm in the laboratory, with its tentacles and gills protruding out of its tube, which is made of artificial sandgrains.

**Which part of this research project was the most rewarding?**

The practical work for this publication was carried out at the Royal Netherlands Institute for Sea Research on the island of Texel. Living on an island and familiarizing myself with a research institute outside my home university was an incredibly enjoyable experience. I have fond memories of working on the experimental setup alongside the technical staff, who shared a wealth of practical knowledge that has proven to be extremely valuable.

**What do you enjoy most about being an early-career researcher?**

The wide range of enjoyable opportunities that arose during and after my studies was truly memorable. While working on my thesis – now partly published in BiO – at the Royal Netherlands Institute for Sea Research, I assisted with fieldwork on an uninhabited island in the Wadden Sea, attended a scientific conference, and taught high school students about my research project. Throughout my studies, I also met many inspiring people, from scientists to climate activists, who shared their passions and motivations and broadened my perspective.

Try to publish your Master's thesis, even if it requires a bit of extra effort

**What piece of advice would you give to the next generation of researchers?**

Try to publish your Master's thesis, even if it requires a bit of extra effort. For me, it was a great way to stay in touch with my supervisors after completing my ECTS and helped me determine whether pursuing an academic career was the right path for me. Above all, it is highly rewarding to showcase the work that my supervisors and I invested in the project

**What's next for you?**

I am currently exploring interesting research topics to focus on as a PhD student. I am keeping my options open by both keeping an eye on vacancies and discussing ideas with scientists to collect my own research funding. Since my interests are broad and I am highly motivated, I am sure an interesting opportunity will arise soon!
